# Powassan Virus in Mammals, Alaska and New Mexico, USA, and Russia, 2004–2007

**DOI:** 10.3201/eid1912.130319

**Published:** 2013-12

**Authors:** Eleanor R. Deardorff, Robert A. Nofchissey, Joseph A. Cook, Andrew G. Hope, Albina Tsvetkova, Sandra L. Talbot, Gregory D. Ebel

**Affiliations:** University of New Mexico, Albuquerque, New Mexico, USA (E.R. Deardorff, R.A. Nofchissey, J.A. Cook);; U S Geological Survey, Anchorage, Alaska, USA (A.G. Hope, S.L. Talbot);; Institute of Biology, Moscow, Russia (A. Tsvetkova);; Colorado State University, Fort Collins, Colorado, USA (G.D. Ebel)

**Keywords:** Powassan virus, deer tick virus, arboviruses, viruses, tick-borne encephalitis, tick-borne encephalitis virus, ecology, Flaviviridae, mammals, Myodes, Peromyscus, serology, zoonoses, Russia, Alaska, New Mexico

## Abstract

Powassan virus is endemic to the United States, Canada, and the Russian Far East. We report serologic evidence of circulation of this virus in Alaska, New Mexico, and Siberia. These data support further studies of viral ecology in rapidly changing Arctic environments.

Powassan virus (POWV) is a tick-borne virus (family *Flaviviridae*, genus *Flavivirus*) with recent and increasing prevalence. The only member of the tick-borne encephalitis (TBE) serogroup of flaviviruses endemic to North America, POWV is an emerging cause of human illness and death (*1**,**2*). Transmitted primarily by *Ixodes* spp. ticks and maintained in enzootic cycles involving small- to medium-size mammals, POWV exists as 2 genetically divergent and spatially distinct lineages that are serologically indistinguishable: lineage I, prototype POWV and lineage II, deer tick virus (DTV) (*3*,*4*). The 2 lineages are maintained in different vector and host species.

First discovered in eastern Canada, POWV is now known to also circulate in the northeastern United States and the Russian Far East and has been documented in the western United States and Canada in wildlife and human infections (*5*–*9*). Clinical signs range from self-limiting febrile illness to severe neurologic disruption and death (*2*). Both lineages have been isolated from persons with fatal cases, and the incidence of human infection increased from an average of 0.7 cases/year (1958–1998) to 1.9 cases/year (1999–2007) (*2*,*10*). This apparent increase, coupled with the relatively recent discovery of lineage II and the well-documented diversity of TBE serogroup flaviviruses in the Old World, highlights the medical role of POWV and related viruses in North America.

Several TBE serogroup viruses, including POWV, also occur in the Russian Far East (*6*). Two hypotheses have emerged regarding the geographic distribution of POWV. The first hypothesis is that TBE serogroup flaviviruses in the Old and New Worlds persisted during the Pleistocene Epoch in Palearctic and Nearctic refugia (refuge areas), respectively, and then spread across continents (*11*). The second hypothesis is that POWV was introduced into Russia from North America in the 20th century (*6*,*12*). These hypotheses are not mutually exclusive, and POWV or closely related TBE serogroup viruses may be endemic to Beringia, the region surrounding the Bering Strait that connects Asia and North America. Because high latitude environments are experiencing rapid rates of change, and the distribution of POWV in North America is unclear, documenting potential sylvatic hosts of this pathogen is critical to evaluating its capacity to emerge into human populations.

The purpose of this study was to better understand the prevalence, distribution, and host specificity of POWV in western North America and Siberia. We also investigated the history and dynamics of POWV or related TBE serogroup viruses in Beringia.

## The Study

**Figure 1:**
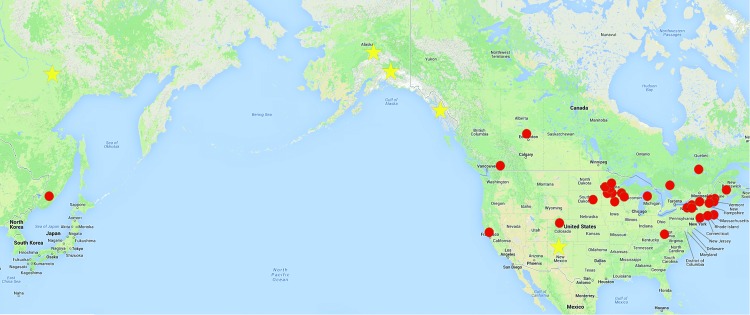
Worldwide distribution of previously confirmed Powassan virus activity. Dots indicate approximate locations of known Powassan virus circulation as shown by human illness, virus isolation from animals, and Powassan virus–specific antibodies in humans or animals. Stars indicate approximate locations of antibody-positive animals, reported herein, collected during 2004–2007 and from whom samples were tested by using a strip immunoblot assay.

Animals were collected in live traps and snap traps from sites in Siberia (2006), Alaska (2004–2005) and throughout the southwestern United States (2005–2007 (Table 1, Figure 1) under University of New Mexico Institutional Animal Care and Use Committee protocol 12–100764-MCC. Blood was collected on site during specimen processing. We screened blood samples from > 600 wild small-to-medium sized mammals representing 31 host species for POWV-specific antibodies. 

**Table 1:** Powassan virus seroprevalence in mammals captured in eastern Russia (Siberia), Alaska, and the southwestern United States*

Region	Species	Common name	No. positive/no. tested (%)	95% CI
Siberia, Russia				
	*Lepus timidus*	Mountain hare	0/1 (0)	0–79.35
	*Microtus gregalis*	Narrow-headed vole	0/2 (0)	0–65.76
	*Microtus oeconomus*	Tundra vole	0/12 (0)	0–24.25
	*Mustela erminea*	Stoat	0/1 (0)	0–79.35
	*Myodes rufocanus*	Gray red-backed vole	0/6 (0)	0–39.03
	*Myodes rutilus*	Northern red-backed vole	6/79 (7.6)	3.52–15.59
	*Myopus schisticolor*	Wood lemming	0/2 (0)	0–65.76
	*Sciurus vulgaris*	Tuft-eared squirrel	0/2 (0)	0–65.76
	*Spermophilus undulatus*	Long-tailed ground squirrel	0/1 (0)	0–79.35
	*Tamias sibiricus*	Siberian chipmunk	0/5 (0)	0–43.45
Total	NA	NA	6/111 (5.4)	NA
Central Alaska				
	*Microtus oeconomus*	Tundra vole	0/5 (0)	0–43.45
	*Mustela vison*	American mink	0/2 (0)	0–65.76
	*Myodes rutilus*	Northern red-backed vole	14/243 (5.8)	3.46–9.44
	*Sorex cinereus*	Cinereus shrew	0/8 (0)	0–32.44
	*Sorex hoyi*	Pygmy shrew	0/1 (0)	0–79.35
	*Sorex monticolus*	Montane shrew	0/6 (0)	0–39.03
	*Sorex tundrensis*	Tundra shrew	0/2 (0)	0–65.76
	*Tamiasciurus hudsonicus*	Red squirrel	0/3 (0)	0–56.15
Southern Alaska				
	*Myodes gapperi*	Southern red-backed vole	6/89 (6.7)	3.13–13.93
Total	NA	NA	20/359 (5.6)	NA
Southwestern USA				
	*Dipodomys merriami*	Merriam's kangaroo rat	0/15 (0)	0–20.39
	*Dipodomys ordii*	Ord's kangaroo rat	0/1 (0)	0–79.35
	*Mus musculus*	House mouse	0/4 (0)	0–48.99
	*Neotoma albigula*	White-throated woodrat	0/10 (0)	0–27.75
	*Neotoma cinerea*	Bushy-tailed woodrat	0/4 (0)	0–48.99
	*Notiosorex crawfordi*	Desert shrew	0/1 (0)	0–79.35
	*Onychomys arenicola*	Mearn's grasshopper mouse	0/14 (0)	0–21.53
	*Perognathus flavus*	Silky pocket mouse	0/3 (0)	0–56.15
	*Peromyscus boylii*	Brush mouse	0/6 (0)	0–39.03
	*Peromyscus eremicus*	Cactus mouse	0/19 (0)	0–16.82
	*Peromyscus maniculatus*	Deer mouse	2/33 (6.0)	1.68–19.61
	*Peromyscus leucopus*	White-footed mouse	0/22 (0)	0–14.87
	*Peromyscus truei*	Piñon mouse	2/9 (22.2)	6.32–54.74
	*Sigmodon hispidus*	Hispid cotton rat	0/3 (0)	0–56.15
TotalSubtotal	NA	NA	4/144 (2.8)	NA

*NA, not applicable.

Serum samples and supernatants were tested by using a strip immunoblot assay (SIA) with recombinant DTV envelope glycoprotein. Because POWV and DTV are serologically indistinguishable, this antigen binds antibodies specific for DTV, POWV, or other closely related viruses.

In brief, antigen was adhered to a nitrocellulose membrane, and strips were produced with the following antigens and markers: Coomassie blue (orientation control), specific pathogen–free mouse serum (negative control), serum from mice inoculated with DTV envelope glycoprotein (DTV-positive control), mouse IgG (IgG-positive control), and purified DTV E-glycoprotein (test). Approximately 0.25 µg of DTV envelope glycoprotein was used per 2-mm test strip. Samples were tested at a 1:200 dilution, and antibody was detected by using an alkaline phosphatase–conjugated secondary antibody (goat antimouse IgG). Colorimetric intensity was assessed and DTV envelope glycoprotein–positive results were compared with 3+ and 1+ IgG control bands.

In Siberia and central Alaska, antibodies reacting with DTV antigen were detected exclusively in northern red-backed voles (*Myodes rutilus*) (6.2%) (Table 1). In southern Alaska, DTV-reactive antibodies were detected in the only species tested, the southern red-backed vole (*M. gapperi*) (6.7%). In the southwestern United States, DTV-reactive antibodies were found in New Mexico in 2 *Peromyscus* species mice: the piñon mouse (*P. truei*) and the deer mouse (*P. maniculatus*) (22.2% and 6.0%, respectively) that were collected sympatrically. The deer mouse is of particular interest because it is the primary host of Sin Nombre virus, the etiologic agent of hantavirus cardiopulmonary syndrome in North America (*13*).

To identify the virus responsible for serologic reactivity, we collected ticks (*Ixodes angustus)* from coastal southeastern Alaska (61.3210°N, 145.3030°W; 59.2459°N, 135.1753°W; and 55.8717°N, 132.3481°W) in 2009 from captured mammals (Table 2). Reverse transcription PCR was performed for ticks and tissues from seronegative animals collected proximally to seropositive animals and thus potentially in the acute stage of infection. No viral RNA was detected in ticks or in seronegative rodent tissue.

**Table 2:** Ticks collected from trapped mammals in southeastern Alaska, USA, June–July 2009, and tested by reverse transcription PCR for flavivirus RNA*

Host species	No.	Adult males	Adult females	Nymphs	Lavae	Total	Average infestation
*Microtus longicaudus*	2	0	1	1	0	2	1.0
*Microtus pennsylvanicus*	1	0	0	1	0	1	1.0
*Myodes gapperi*	18	1	17	33	4	55	3.1
*Myodes rutilus*	12	0	5	9	2	16	1.3
*Peromyscus keeni*	21	2	16	33	26	77	3.7
*Peromyscus maniculatus*	5	0	2	3	0	5	1.0
*Sorex cinereus*	3	0	3	12	0	15	5.0
*Sorex monticolus*	10	0	0	18	22	40	4.0
*Synaptomys borealis*	1	0	0	10	0	10	10.0
*Tamiasciurus hudsonicus*	6	0	8	2	2	12	2.0
Total	79	3	52	122	56	233	2.9

*Several individual ticks (1 adult male, 3 adult females, and 12 nymphs) were not tested by reverse transcription PCR because of desiccation during storage. No larvae were tested. Infestation rate was calculated by dividing the total number of ticks by the total number of individuals for each mammalian species.

## Conclusions

Although we used a DTV antigen because of its technical convenience, we do not believe that DTV per se is present in these rodent populations. POWV is present throughout western United States and western Canada. However, the virus responsible for the observed seropositivity in Alaska is unknown. The most likely candidate is POWV but without an isolate or sequence data, tick-borne encephalitis virus or other Eurasian flavivirus cannot be ruled out, and we cannot rule out the possibility that the virus is a flavivirus with no known vector. The utility of the SIA is partially based on known cross-reactivity of flaviviruses because it enables detection of divergent lineages. Determination of endpoint antibody titers and confirmation of POWV specificity by plaque-reduction neutralization tests were not possible because of freezer failure.

Because few wild rodent antibodies are commercially available, our methodology used anti-*Mus* secondary antibody, which may have varying sensitivity against the 31 species tested. Thus, low-level reactivity may have been missed. However, the prevalence of antibodies detected by SIA in our study is consistent with that reported from known POWV transmission foci (*14*).

**Figure 2:**
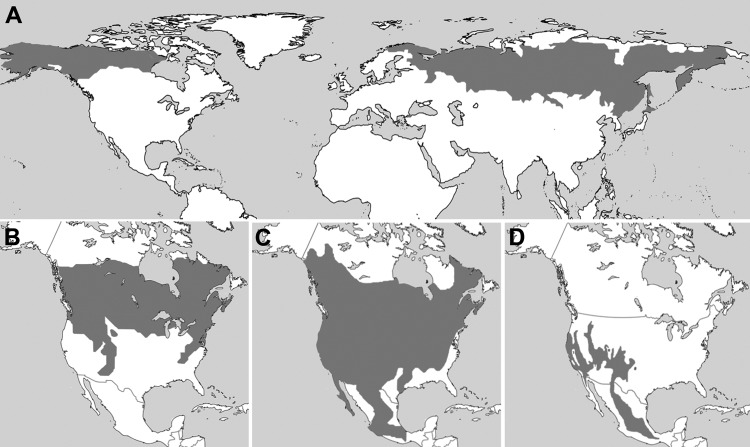
Ranges (gray) of A) northern red-backed vole (*Myodes rutilus*), B) southern red-backed vole (*M. gapperi*), C) deer mouse (*Peromyscus maniculatus*), and D) piñon mouse (*P. truei*), United States, Russia, and Canada. Major range overlap between the 4 species found with deer-tick virus–reactive antibodies suggests that the responsible virus may have access to competent amplifying hosts throughout North America. Panel A was based on the International Union for Conservation of Nature and Natural Resources Red List (www.iucnredlist.org/) and panels B–D were based on the Smithsonian National Museum of Natural History, North American Mammals (www.mnh.si.edu/mna/main.cfm).

These serologic results enable us to conclude that ≥1 flaviviruses antigenically similar to DTV circulate in Siberia, Alaska, and the southwestern United States (Table 1). Transmission appears to involve *Myodes* spp. voles in northern regions and *Peromyscus* mice in southern regions. Considerable overlap in the geographic ranges of these species may provide continuous populations of competent amplifying hosts from Mexico (*P. maniculatus and P. truei*) to Siberia (*M. rutilus*) (Figure 2). The seropositivity in Siberia may be from introduced POWV, native TBE virus, or other related virus. Viral RNA sequence is necessary to delineate the viral species that are circulating among *M. rutilus* in Siberia. Additional host species may be involved; considering the small sample for the current study, seropositivity rates and distributions, although consistent with expectations, may be considerably refined with increased sampling (Table 1). The incidence and host association of *Ix. angustus* ticks were similar to those of a previous report (*15*), and further vectorial studies are warranted.

Our findings augment knowledge of distribution of TBE serogroup flavivirus in the Nearctic and will guide further studies of New World TBE serogroup flavivirus ecology. Future work will focus on acquisition of viral isolates and nucleic acid sequences from *Myodes* spp. voles in Alaska and Siberia and from *Peromyscus* spp. mice in the southwestern United States.
